# Acceptability of a COVID-19 pre-exposure prophylaxis trial with hydroxychloroquine in French healthcare workers during the first wave of COVID-19 pandemic

**DOI:** 10.1186/s13063-021-05329-y

**Published:** 2021-05-30

**Authors:** Amandine Gagneux-Brunon, Clémentine Schilte, Arnauld Garcin, Nathalie Jolly, Muriel Vray, Laura Schaeffer, Xavier Duval, Bruno Hoen, Elisabeth Botelho-Nevers

**Affiliations:** 1 Department of Infectious diseases, University Hospital of Saint-Etienne, Saint-Etienne, 42055 France; 2 CIC-1408 Vaccinology, INSERM, University Hospital of Saint-Etienne, Saint-Etienne, 42055 France; 3 Center for Translational Science, Institut Pasteur, Paris, France; 4 Unité de Recherche Clinique, Innovation et Pharmacologie, University Hospital of Saint-Etienne, Saint-Etienne, 42055 France; 5 Unité d’Epidémiologie des Maladies Emergentes, Institut Pasteur, INSERM, Paris, France; 6 Inserm CIC-1425, AP-HP, Hôpital Universitaire Bichat; Inserm UMR-1137 IAME; Université Paris Diderot, UFR de Médecine-Bichat, Paris, France; 7 Institut Pasteur, Paris, France

**Keywords:** Pre-exposure chemoprophylaxis trial, SARS-CoV-2 infection, Healthcare workers, Acceptability, Hydroxychloroquine

Healthcare workers (HCWs) have been over-represented among people infected with Severe Acute Respiratory Syndrome-Coronavirus-2 (SARS-CoV-2). In France, between March 1 and November 2, 2020, SARS-CoV-2 infection was diagnosed in more than 44,000 HCWs and killed 17 [1]. In the USA, HCWs accounted for around 20% of the confirmed cases of SARS-CoV-2 infections by April 2020 [2]. Protection of HCWs rapidly became a crucial challenge during the COVID-19 pandemics [3]. Pre- and post-exposure chemoprophylaxis was considered to be used in addition to personal protective equipment. More than 50 clinical trials aiming to assess chloroquine or hydroxychloroquine (HCQ) in HCWs and/or household contacts of COVID-19 cases have been registered worldwide with ClinicalTrials.gov. In France in April 2020, a multi-center randomized, double-blind, placebo-controlled trial to evaluate the efficacy of a 2-month pre-exposure prophylaxis with HCQ in 600 HCWs exposed to COVID-19 patients was started [4]. This trial was funded by the French hospital program for clinical research (PHRC) and sponsored by the University Hospital of Saint-Etienne in collaboration with Institut Pasteur in Paris. For each participant, six visits at the investigation center, one weekly electrocardiogram, 5 blood samplings, and 4 nasopharyngeal swabs for SARS-CoV-2 RT-PCR were scheduled. Before the trial started, we conducted an anonymous online survey in ten of the investigation centers to evaluate the acceptability of the trial among HCWs, whether they worked in a hospital, an ambulatory setting, or a long-term care facility. Eight hundred and seventy-one HCWs completed the survey. Among respondents, 695 (79.8%) reported they were interested in participating in the trial. Intention to participate was not influenced by age or working place. Among the 695 potential volunteers, 430 (61.9%) were under 45 years of age and only 126 (18.1%) reported a significant comorbidity. The main reasons for declining participation in the study were the following: (1) fears about HCQ side effects in 121 of the 176 decliners (68.8%), (2) the perception that the individual risk of severe COVID-19 was low in 36 (20.5%), (3) the constraints resulting from the number of study visits in 29 (16.5%), and (4) the burden of nasopharyngeal swabs for SARS-CoV-2 RT-PCR in 25 (14.2%). Six hundred and ninety-five respondents reported being interested in participating in the chemoprophylaxis trial, and 117 were actually enrolled by May 27, 2020. In the first center where the trial was started, 342 HCWs reported being interested in participating in the trial and 91 were enrolled between April 14 and May 27 2020, when the French Medicine Agency put all trials evaluating HCQ on hold.

After the French Medicine Agency’s decision and as the French epidemic curve was decreasing and the recruitment became challenging, the trial was stopped. This work shows that, at the time the survey was launched, French HCWs were prone to participate in a SARS-CoV-2 infection chemoprophylaxis trial with HCQ, although fear of side effects was the primary reason to decline study participation. This information was key in our decision to start the trial.

After two randomized, double-blind, placebo-controlled trials showed no protective effect of HCQ in HCWs exposed to SARS-CoV-2 [5, 6], it is now admitted that HCQ is unlikely to act as a prophylactic agent against COVID-19. However, the evaluation of other agents and regimens would still be of interest in high-risk people until effective vaccines are widely available. Given the acceptability rate we observed in our survey, the feasibility of clinical trials on chemoprophylaxis of COVID-19 in HCWs appears reasonably fair. Nevertheless, we suggest that before starting trials involving hundreds of volunteers, surveys need be conducted to assess participants' acceptability and preferences among preventive strategies to be tested.

## Data Availability

EBN and AGB have access to the final database of acceptability study reported here.
